# Putting the National Diabetes Prevention Program to Work: Predictors of Achieving Weight-Loss Goals in an Employee Population

**DOI:** 10.5888/pcd16.190053

**Published:** 2019-09-12

**Authors:** Rosette J. Chakkalakal, Lisa R. Connor, Lori A. Rolando, Yi Huang, Daniel W. Byrne, Bradley M. Awalt, Paula W. McGown, Muktar H. Aliyu, Mary I. Yarbrough

**Affiliations:** 1 Department of Medicine, Vanderbilt University Medical Center, Nashville, Tennessee; 2 Center for Diabetes Translation Research, Vanderbilt University Medical Center, Nashville, Tennessee; 3 Vanderbilt Health and Wellness, Vanderbilt University Medical Center, Nashville, Tennessee; 4 Department of Biostatistics, Vanderbilt University Medical Center, Nashville, Tennessee; 5 Department of Health Policy, Vanderbilt University Medical Center, Nashville, Tennessee

## Abstract

**Introduction:**

Differences in eligibility criteria and intervention characteristics have limited the generalizability of findings from studies of worksite translations of the National Diabetes Prevention Program (DPP). The objective of our study was to identify factors associated with achievement of the DPP’s 5% weight-loss goal in the Vanderbilt University Medical Center (VUMC) Faculty and Staff Health and Wellness DPP from 2014 to 2017.

**Methods:**

We analyzed data from a DPP worksite translation that adhered to national standards for program quality and intervention fidelity. We compared baseline characteristics and program metrics for participants who did and did not achieve the program’s 5% weight-loss goal, and we developed a multivariable logistic regression model to identify independent predictors of achieving this goal.

**Results:**

Of the 165 employees enrolled in the DPP from 2014 to 2017, 43.6% (n = 72) met the 5% weight-loss goal. Mean (standard deviation) percentage weight loss for the program was 5.2% (6.0%), or 4.8 (6.0) kg. The median (interquartile range) body mass index at baseline was lower among participants who achieved the 5% weight-loss goal than among those who did not (31.6 [29.4–37.4] vs 34.7 [31.5–39.2], *P* = .009), and participants who achieved the goal reported more physical activity minutes per week (166.0 [135.2–223.0] min vs 128.5 [83.2–169.8] min, *P* < .001). Session attendance was greater for participants achieving the 5% weight-loss goal (23 [21–25]) sessions vs 18 [12–21] sessions, *P* < .001). In the adjusted analysis, physical activity and session attendance remained significant predictors of achieving the 5% weight-loss goal.

**Conclusion:**

Session attendance and physical activity independently predicted achievement of the 5% weight-loss goal in this worksite translation of the DPP. Strategies designed to improve these metrics may increase DPP success rates.

**Summary**

**What is already known on this topic?**

Worksites are valuable ancillary health care systems for population health promotion efforts, particularly the National Diabetes Prevention Program (DPP). Differences in key program characteristics have, however, limited the generalizability of findings from studies of worksite translations of the DPP.

**What is added by this report?**

We evaluated the effectiveness of the Vanderbilt University Medical Center Faculty and Staff Health and Wellness DPP, a worksite translation of the DPP that earned full recognition status from the Centers for Disease Control and Prevention in 2017.

**What are the implications for public health practice?**

Increased session attendance and increased physical activity among participants may increase success rates for employer-based DPPs.

## Introduction

Worksites provide ideal settings to disseminate evidence-based health promotion programs. Sixty percent of US adults 16 years or older are employed (1), and worksites are a key source of information, communication, and support for employees (2). The substantial effect of obesity on health care costs, productivity, absenteeism, and disability, has created financial incentives to focus worksite wellness efforts on obesity (3). Employers spend 37% more on health care for obese adults than for normal-weight adults; most of this excess expenditure is attributable to type 2 diabetes, hyperlipidemia, and heart disease (4).

In 2010 the US Congress authorized the Centers for Disease Control and Prevention (CDC) to establish the National Diabetes Prevention Program (DPP). The DPP is based on data from several randomized controlled trials (5) demonstrating that type 2 diabetes can be prevented or delayed in adults at high risk through a structured lifestyle intervention (6). Targeted efforts by CDC, the American Diabetes Association, the American Medical Association, and the National Business Coalition on Health (7) have resulted in more than 60 employers and insurers now offering the DPP as an evidence-based weight-management program to employees (8).

Several studies evaluated DPP implementation efforts in community and clinical settings (9), but few focused on DPP implementation at worksites (7). Differences in program delivery limited the generalizability of findings from previous DPP worksite translation studies (7). The CDC Diabetes Prevention Recognition Program (DPRP) was established to minimize differences in program delivery by ensuring program quality and fidelity to scientific evidence (10–12). The objective of our study was to identify factors associated with achievement of the DPP’s 5% weight-loss goal in the Vanderbilt University Medical Center (VUMC) Faculty and Staff Health and Wellness DPP, a worksite translation of the DPP that earned full recognition status from the CDC DPRP in 2017.

## Methods

Health Plus, the workplace wellness division of Vanderbilt Health and Wellness, began offering the VUMC Faculty and Staff Health and Wellness DPP to the approximately 25,000 employees of Vanderbilt University (VU) and VUMC in 2014 (at no cost to employees). The eligibility criteria for the VUMC Faculty and Staff Health and Wellness DPP adhered to the 2015 DPRP standards for DPP participant eligibility (12). VU/VUMC employees had to be aged 18 or older and have a body mass index (BMI, in kg/m^2^) of 24.0 or more (≥22.0, if Asian American). Additionally, employees had to meet at least 1 of the following criteria to qualify for the program: 1) a blood test result within the previous year consistent with a diagnosis of prediabetes (fasting glucose of 100–125 mg/dL, plasma glucose measured 2 hours after a 75g glucose load of 140–199 mg/dL, or hemoglobin A_1C_ of 5.7%–6.4%), 2) clinically diagnosed gestational diabetes mellitus during a previous pregnancy, or 3) a positive screening result on the American Diabetes Association or CDC questionnaires for prediabetes (10,13,14). People who were pregnant at the time of enrollment or had been diagnosed with type 1 or type 2 diabetes before enrollment were not eligible.

### The VUMC Faculty and Staff Health and Wellness DPP

Consistent with the focus of the National DPP, the VUMC Faculty and Staff Health and Wellness DPP helps participants make moderate changes in diet and physical activity to achieve modest weight loss (5%–7% of baseline body weight) by presenting information, providing outside-of-class activities, and offering feedback to optimize behavior change (10,12). The program emphasizes self-monitoring of diet and physical activity, building self-efficacy and social support for maintaining behavior changes, and problem-solving strategies for overcoming common challenges to sustaining weight loss. The VUMC Faculty and Staff Health and Wellness DPP follows the 2012 National DPP curriculum (15), which was approved by CDC as meeting the requirements of the DPRP (10,12). Classes were delivered in person in a group setting and were organized into 16 core sessions during the first 6 months of the program, followed by 6 to 10 postcore sessions (per CDC guidance) during the second 6 months of the program.

### Study design

We conducted an exploratory analysis of data from DPP participants enrolled in the first 5 cohorts (June 24, 2014, through August 28, 2017) of the VUMC Faculty and Staff Health and Wellness DPP. In accordance with the 2015 DPRP standards for evaluating DPP outcomes, we categorized people as DPP participants if they attended at least 4 sessions during the 12-month program (12). The primary analyses focused on the comparison of baseline characteristics and program metrics for DPP participants who achieved the minimum 5% weight-loss goal and those who did not. Through a Research Electronic Data Capture (REDCap) survey (16) at the time of enrollment, participants were asked to self-report their age, sex, and race/ethnicity, and indicate the method by which they qualified for the DPP (ie, blood test, questionnaire, or diagnosed gestational diabetes, or various combinations thereof). We calculated baseline BMI by using height and weight data collected from participants at the first DPP session. Program characteristics were physical activity minutes per week, the number of sessions attended, and weight loss. Physical activity was self-reported by DPP participants at each DPP session. We assessed weekly physical activity minutes by asking participants to respond to the following question for each session via REDCap survey: “Please report your total physical activity minutes for the past week. The minutes you report should be moderate intensity, meaning you are going fast enough to breathe heavier than usual, but not so fast that you are unable to talk. An example of this is brisk walking. Report any activity that you have done for at least 10 minutes or longer.” Participants were not required to complete the REDCap survey to report their weekly physical activity minutes if they instead verbally reported physical activity minutes to their coach or submitted this data via email as a free text report. We calculated an overall mean physical activity minutes per week for each participant. Health Plus staff members measured participants’ weight at each DPP session. Health Plus staff members are trained by the Health Plus nurse case manager to follow a standardized protocol when measuring height and weight; protocol proficiency is re-assessed annually. Per the CDC DPRP protocol for calculating participants’ percentage change in weight (10), we categorized participants as having achieved the 5% weight-loss goal (yes or no) after calculating each participant’s percentage weight loss (difference in weight between the first and last session attended divided by baseline weight, then multiplied by 100).

The Vanderbilt Institutional Review Board recognized this evaluation as a quality improvement project for the purposes of evaluating program efficacy, quality improvement, and dissemination of program results.

### Statistical analysis

We used Fisher exact tests for categorical variables and Wilcoxon rank-sum tests for continuous and ordinal variables to compare baseline characteristics and program metrics between DPP participants who achieved the 5% weight-loss goal and participants who did not. For categorical variables, we calculated the success rate by dividing the number of DPP participants who achieved the 5% weight-loss goal by the total number of participants in each category. We used LOWESS (locally weighted scatterplot smoothing) nonparametric regression trend lines with 95% confidence intervals to display percentage weight change during the program. These spline graphs provide a nonlinear smoothed curve based on a moving average to find a curve of best fit without assuming the data must fit some distribution shape. Baseline characteristics and program metrics that were significantly associated with achievement of the 5% weight-loss goal in bivariate analyses were included in a logistic regression model to determine which variables remained significant predictors of the 5% weight-loss goal. For ease of interpretation, we analyzed physical activity as 30-minute intervals per week in the logistic regression model. All analyses were performed using R version 3.4.3 (The R Foundation) (17).

## Results

During the study period, 165 employees enrolled in the VUMC Faculty and Staff Health and Wellness DPP. The mean (standard deviation [SD]) age of DPP participants was 50.3 (8.6) years, and 85% were women. Most (77%) participants were obese, 64% of participants were non-Hispanic white, and 26% were non-Hispanic black. The general VU/VUMC employee population in 2017 was younger (mean 43.0 [SD, 12.8] y) than the DPP participant population, and a smaller percentage (27%) was obese. These differences reflect eligibility criteria for the DPP, because the likelihood that a person will have a positive screen for prediabetes on either the American Diabetes Association or CDC prediabetes questionnaires increases with increasing age and/or increasing BMI.

**Figure 1:**
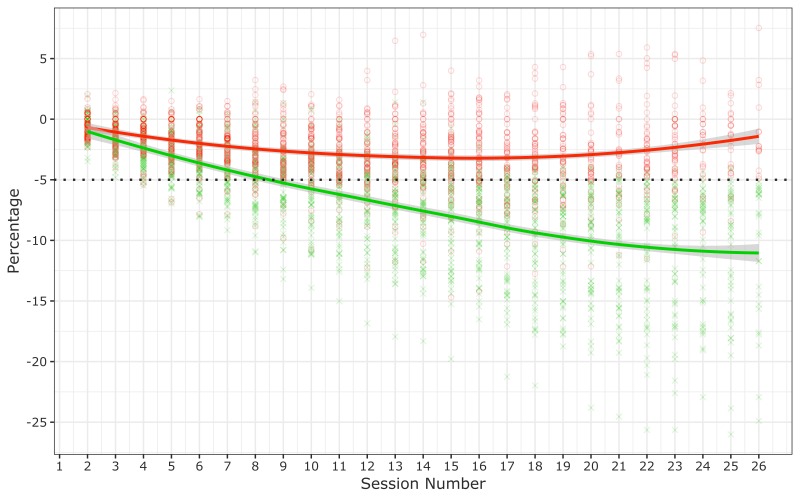
Percentage change in weight among 165 participants in the Vanderbilt University Medical Center (VUMC) Faculty and Staff Health and Wellness Diabetes Prevention Program, 2014–2017. The dotted line represents the 5% weight-loss goal. Each green cross represents a participant who achieved the 5% weight-loss goal. Each red circle represents a participant who did not achieve the 5% weight-loss goal. The solid red line and the solid green line are LOWESS (locally weighted scatterplot smoothing) nonparametric regression trend lines; shading indicates 95% confidence intervals.

Of the 165 participants, 72 (43.6%) employees met the 5% weight-loss goal (Table 1). Mean (SD) percentage weight loss for the full cohort was 5.2% (6.0%), or 4.8 (6.0) kg. The trend line for participants who met the target weight-loss goal crossed the 5% weight-loss threshold at approximately session 9 (Figure 1). Participants achieving the 5% weight-loss goal lost a median (interquartile range [IQR]) 7.5 (5.3–13.3) kg, or 8.0% (6.2%–13.9%) of baseline weight; participants who did not achieve the goal lost a median (IQR) 1.4 (0–3.2) kg (Table 2).

**Table 1:** Baseline Characteristics of DPP Participants (N = 165) by Achievement of 5% Weight-Loss Goal, Vanderbilt University Medical Center Faculty and Staff Health and Wellness DPP, 2014–2017

Characteristic	5% Weight-Loss Goal Not Met^a^	5% Weight-Loss Goal Met^a^	Success Rate, %^b^	*P* Value
**No. of participants**	93	72	43.6	—
**Age, median (IQR), y**	51.0 (44.0–56.0)	52.0 (47.0–57.0)	—	.23^c^
**Sex**				
Female	80 (86.0)	60 (83.3)	42.9	.67^d^
Male	13 (14.0)	12 (16.7)	48.0	
**Race/ethnicity**				
Non-Hispanic white	60 (64.5)	45 (62.5)	42.9	.74^d^
Non-Hispanic black	24 (25.8)	18 (25.0)	42.9	
Hispanic	4 (4.3)	3 (4.2)	42.9	
Non-Hispanic Asian	4 (4.3)	4 (5.6)	50.0	
Other or unknown	1 (1.1)	2 (2.8)	66.7	
**BMI, median (IQR)**	34.7 (31.5–39.2)	31.6 (29.4–37.4)	—	.009^c^
**BMI category, kg/m^2^**				
Normal (18.5–24.9)	1 (1.1)	3 (4.2)	75.0	.04^d^
Overweight (25.0–29.9)	14 (15.1)	20 (27.8)	58.8	
Obese (≥30.0)	78 (83.9)	49 (68.1)	38.6	
**DPP qualification method^e^**				
Blood test	1 (1.1)	1 (1.4)	50.0	.40^d^
Questionnaire	41(44.1)	20 (27.8)	32.8	
Diagnosed gestational diabetes	1 (1.1)	1 (1.4)	50.0	
Blood test and questionnaire	35 (37.6)	38 (52.8)	52.1	
Blood test and diagnosed gestational diabetes	2 (2.2)	1 (1.4)	33.3	
Diagnosed gestational diabetes and questionnaire	10 (10.8)	8 (11.1)	44.4	
Blood test, diagnosed gestational diabetes, and questionnaire	3 (3.2)	3 (4.2)	50.0	

Abbreviation: BMI, body mass index; DPP, Diabetes Prevention Program; IQR, interquartile range.

a Values are number (percentage) unless otherwise indicated.

b Success rate calculated by dividing the number of DPP participants who achieved the 5% weight-loss goal by the total number of participants in each category.

c *P* value based on the nonparametric Wilcoxon rank-sum test.

d *P* value based on the Fisher exact test.

e To qualify to participate in the program, employees had to meet ≥1 of the following criteria: 1) a blood test result within the previous year consistent with a diagnosis of prediabetes (fasting glucose of 100–125 mg/dL, plasma glucose measured 2 hours after a 75-g glucose load of 140–199 mg/dL, or hemoglobin A_1C_ of 5.7%–6.4%), 2) clinically diagnosed gestational diabetes mellitus during a previous pregnancy, or 3) a positive screening result on a questionnaire for prediabetes (10,13,14). In addition, employees had to be aged ≥18 and have a BMI ≥24.0 (≥22.0, if Asian American).

**Table 2:** Program Metrics for DPP Participants (N = 165) by Achievement of 5% Weight-Loss Goal, Vanderbilt University Medical Center Faculty and Staff Health and Wellness DPP, 2014–2017^a^

Metric	5% Weight-Loss Goal Not Met^b^	5% Weight-Loss Goal Met^b^	*P* Value^c^
**No. of participants**	93	72	—
**Physical activity, minutes per week**			
Core phase	132.0 (91.5–164.0)	163.5 (132.8–220.5)	<.001
Postcore phase	120.0 (62.5–173.0)	165.0 (119.0–231.0)	<.001
Overall program	128.5 (83.2–169.8)	166.0 (135.2–223.0)	<.001
**No. of sessions of attended**			
Core phase	14 (12–15)	15 (14–15)	<.001
Postcore phase	4 (0–7)	8 (6–10)	<.001
Overall program	18 (12–21)	23 (21–25)	<.001
**Last session attended^d^**	23 (16–25)	25 (23–26)	<.001
**Percentage of weight loss**			
Core phase	2.5 (0.6–4.4)	7.2 (5.7–10.3)	<.001
Overall program	1.3 (0–3.3)	8.0 (6.2–13.9)	<.001
**Absolute weight loss**, **kg**			
Core phase	2.3 (0.9–4.1)	6.4 (5.0–8.7)	<.001
Overall program	1.4 (0–3.2)	7.5 (5.3–13.3)	<.001

Abbreviation: DPP, Diabetes Prevention Program.

a Classes were delivered in person in a group setting and were organized into 16 core sessions during the first 6 months of the program, followed by 6 to 10 postcore sessions during the second 6 months of the program.

b Values are median (interquartile range) unless otherwise indicated.

c *P* value for the nonparametric Wilcoxon rank-sum test.

d Of the sessions offered, numbered sequentially from 1 to 26, the last session attended.

Participants who achieved the 5% weight-loss goal were more likely than those who did not achieve the goal to have a lower baseline BMI (median [IQR] 31.6 [29.4–37.4] vs 34.7 [31.5–39.2]; *P* = .009). We found no significant differences in achievement of the 5% weight-loss goal by age, sex, race/ethnicity, or qualification method. The most common program qualification method was the combination of a positive screening questionnaire and a blood test in the prediabetes range in the previous year (73 of 165 participants, or 44.2%) (Table 1). Although qualification method was not significantly associated with achievement of the 5% weight-loss goal, success rates were lower for participants who qualified solely on the basis of a positive screening questionnaire (32.8% success rate) than for participants who qualified on the basis of the combination of a positive screening questionnaire and a blood test (52.1% success rate).

**Figure 2:**
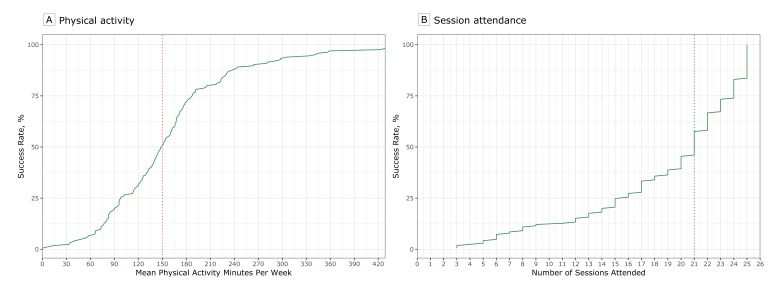
Success rates for achievement of 5% weight-loss goal among 165 participants in the Vanderbilt University Medical Center (VUMC) Faculty and Staff Health and Wellness Diabetes Prevention Program, 2014–2017. The red dotted line (A) indicates 150 minutes of physical activity and the blue dotted line (B) indicates 21 sessions. The points at which the red and blue dotted lines intersect with the solid green line indicate 50% success rates.

We observed significant differences in all program metrics when we compared participants who achieved the 5% weight-loss goal and those who did not (Table 2). Participants who achieved the 5% weight-loss goal reported a median [IQR] 166.0 [135.2–223.0] physical activity minutes per week, whereas participants who did not achieve goal reported 128.5 [83.2–169.8] physical activity minutes per week (*P* < .001). Similarly, participants who achieved the 5% weight-loss goal attended more program sessions than those who did not meet the weight-loss goal (23 [21–25] vs 18 [12–21] sessions; *P* < .001). These findings were consistent in both the core and postcore phases of the program. The median for the last DPP session attended was session 25 for participants who achieved the 5% weight-loss goal and session 23 for participants who did not achieve the goal (*P* < .001). Participants who reported an average of at least 150 minutes of physical activity per week or attended at least 21 DPP sessions had a 50% success rate in achieving the 5% weight loss goal (Figure 2). The steep slope of the line indicates that increasing physical activity above 150 minutes was associated with significantly higher success rates. Similarly, attending 21 sessions was associated with a 50% success rate, but the slope of the line indicates that increasing attendance to more than 21 sessions was associated with much higher success rates.

Baseline BMI, physical activity, and the number of sessions attended differed significantly between participants who achieved the 5% weight-loss goal and participants who did not, so we included these variables in the logistic regression model. In the adjusted analysis, only physical activity and number of sessions attended remained significant predictors of achieving the 5% weight-loss goal. The odds of achieving the 5% weight-loss goal were 20% greater for every additional 30-minute interval of physical activity per week (odds ratio [OR] = 1.20, 95% confidence interval [CI], 1.02–1.41; *P* = .02); the odds of achieving the 5% weight loss goal were also 20% greater for every additional session attended (OR = 1.20; 95% CI, 1.10–1.32; *P* < .001). Baseline BMI was not a significant predictor of achieving the goal (OR = 0.97; 95% CI, 0.91–1.04; *P* = .37).

## Discussion

We found that number of sessions attended and weekly minutes of physical activity were independently associated with achieving the 5% weight-loss goal in the VUMC Faculty and Staff Health and Wellness DPP. As a worksite translation of the National DPP with full recognition status from CDC, our program maintains rigorous standards for program quality and fidelity to scientific evidence. Previous studies of DPP worksite translations demonstrated substantial differences in fundamental elements of the DPP, including participant eligibility criteria and intervention characteristics (7). For example, a recent review of translational workplace DPP-based interventions showed that none of the 10 programs included in the review used DPRP’s standard eligibility criteria (7). Only 2 programs offered both the 16-session core phase and the 6-month maintenance phase of the DPP, and 4 programs did not offer any maintenance sessions. The results of our study are more generalizable to other programs participating in the DPRP than findings from previous evaluations of employer-based DPPs because of our adherence to DPRP standards for implementation.

The weight-loss results we observed exceeded the weight-loss results reported in previous studies. Participants in our DPP lost, on average, 5.2% of their body weight at the time of program completion and 43.6% achieved the 5% weight-loss goal. A recent systematic review of real-world translations of the DPP reported a mean weight loss of 4% at 12-month follow-up across the 28 studies included in the analysis (18). A recent assessment of participant-level results from the National DPP found that average weight loss was 4.2% and that 35.5% of participants achieved the 5% weight-loss goal (11). Our results demonstrating the importance of the number of sessions attended in achieving weight loss is consistent with the results of these large multisite evaluations of the DPP (11,18). Ali et al found that for every additional lifestyle session attended, weight loss increased by 0.26% (18). Ely et al similarly found that for every additional DPP session attended, participants lost 0.31% of their body weight (11). Our finding that physical activity is a significant predictor of achieving the 5% weight-loss goal was also consistent with reports by Ely et al, who found that National DPP participants lost 0.3% of their body weight for every 30 additional minutes per week of physical activity reported (11). The consistent identification of the number of sessions attended and physical activity minutes as significant predictors of weight loss in the DPP provides strong evidence that strategies designed to increase session attendance and increase physical activity among DPP participants could increase success rates, particularly in employer-based programs.

Despite the program’s effectiveness, participation rates for our employer-based DPP were low. The Diabetes Prevention Impact Toolkit recently developed by CDC in collaboration with RTI International (19,20) can be used to project the percentage of a population eligible to participate in a DPP based on the unique demographic characteristics of that population. Using this toolkit, we estimated 7,869 of the 25,444 benefits-eligible employees working at VU/VUMC in 2017 would be eligible for the VUMC Faculty and Staff Health and Wellness DPP. Yet only 229 employees completed the DPP in the 4 years it has been available as a benefit. Employer-based health promotion programs frequently report limited program participation (21,22) because of such barriers as geography, inconvenient locations, time limitations, insufficient incentives, and confidentiality concerns (23,24). Limited program participation is a problem within and beyond worksites. A systematic review of “real-world” translations of the DPP reported low participation rates (≤33%) in 25 of 35 studies (25). The rates were 10% or less in half of the studies (25). A recent analysis of National Health Interview Survey data also noted low DPP participation rates; only 2.4% of eligible adults in the sample had participated in the program (26). Feedback collected from our DPP participants during 2014–2017 suggested that the requirement to meet in person on the VU/VUMC main campus was a barrier to participation. We introduced an option to participate in a video-teleconference group (ie, telehealth) in 2018 to overcome this geographic barrier to program participation; future analyses will evaluate whether this option improves program participation rates.

Our study has several limitations. Like previous evaluations of the DPP, our study was limited to analyzing standard programmatic data available for the DPP and did not account for potential unmeasured confounders, including sleep, dietary intake, readiness for behavior change, and others. Physical activity minutes were self-reported and may have been prone to recall bias. Our calculation of weight loss as the difference between the first and last session attended is consistent with DPRP standards (10,12), but it does not account for the fact that the last session may have been earlier than 12 months after the participant enrolled in the DPP. Importantly, the median for the last session attended by participants in our program was 24, suggesting that the last weight recorded for most participants was close to the end of the 12-month program. Women were overrepresented in our sample; 67% of VU/VUMC employees are female but 85% of DPP participants were female. This selection bias is consistent with previous studies of workplace wellness programs, which observed higher participation rates among female employees (27,28).

Worksites are valuable ancillary health care systems for population health promotion efforts among US adults. Addressing weight management at a population level is challenging because a single intervention is unlikely to account for the diverse needs and preferences of so many people. VUMC Faculty and Staff Health and Wellness uses the AMSO framework (Awareness, Motivation, Skill-Building, Opportunity) for workplace health promotion to provide a variety of weight-management options to accommodate differences in readiness for behavior change, availability, goals, degree of support, and other factors among VU/VUMC employees (29). Within this framework, the DPP provides an excellent evidence-based skill-building program option for employees at high risk for developing diabetes. Strategies designed to improve program attendance and increase physical activity among DPP participants may increase success rates for employer-based DPPs adhering to DPRP standards.
